# Equity in antenatal care quality: an analysis of 91 national household surveys

**DOI:** 10.1016/S2214-109X(18)30389-9

**Published:** 2018-10-12

**Authors:** Catherine Arsenault, Keely Jordan, Dennis Lee, Girmaye Dinsa, Fatuma Manzi, Tanya Marchant, Margaret E Kruk

**Affiliations:** a Department of Global Health and Population, Harvard T H Chan School of Public Health, Boston, MA, USA; b Department of Public Health Policy and Management, NYU College of Global Public Health, New York University, New York, NY, USA; c Department of Public Health and Health Policy, College of Health Sciences, Haramaya University, Harar, Ethiopia; d Ifakara Health Institute, Dar es Salaam, Tanzania; e Department of Disease Control, London School of Hygiene & Tropical Medicine, London, UK

## Abstract

**Background:**

Emerging data show that many low-income and middle-income country (LMIC) health systems struggle to consistently provide good-quality care. Although monitoring of inequalities in access to health services has been the focus of major international efforts, inequalities in health-care quality have not been systematically examined.

**Methods:**

Using the most recent (2007–16) Demographic and Health Surveys and Multiple Indicator Cluster Surveys in 91 LMICs, we described antenatal care quality based on receipt of three essential services (blood pressure monitoring and urine and blood testing) among women who had at least one visit with a skilled antenatal-care provider. We compared quality across country income groups and quantified within-country wealth-related inequalities using the slope and relative indices of inequality. We summarised inequalities using random-effects meta-analyses and assessed the extent to which other geographical and sociodemographic factors could explain these inequalities.

**Findings:**

Globally, 72·9% (95% CI 69·1–76·8) of women who used antenatal care reported blood pressure monitoring and urine and blood testing; this number ranged from 6·3% in Burundi to 100·0% in Belarus. Antenatal care quality lagged behind antenatal care coverage the most in low-income countries, where 86·6% (83·4–89·7) of women accessed care but only 53·8% (44·3–63·3) reported receiving the three services. Receipt of the three services was correlated with gross domestic product per capita and was 40 percentage points higher in upper-middle-income countries compared with low-income countries. Within countries, the wealthiest women were on average four times more likely to report good quality care than the poorest (relative index of inequality 4·01, 95% CI 3·90–4·13). Substantial inequality remained after adjustment for subnational region, urban residence, maternal age, education, and number of antenatal care visits (3·20, 3·11–3·30).

**Interpretation:**

Many LMICs that have reached high levels of antenatal care coverage had much lower and inequitable levels of quality. Achieving ambitious maternal, newborn, and child health goals will require greater focus on the quality of health services and their equitable distribution. Equity in effective coverage should be used as the new metric to monitor progress towards universal health coverage.

**Funding:**

Bill & Melinda Gates Foundation.

## Introduction

Although substantial progress has been made in increasing access to health services in low-income and middle-income countries (LMICs), the quality of care provided across different countries and health conditions remains low and hinders progress in improving health outcomes. 1, 2

Good-quality antenatal care is crucial for the prevention and detection of potential causes of obstetric complications and to avert newborn deaths and stillbirths.^3^, ^4^ For example, without adequate treatment, it is estimated that more than half of pregnancies in women with syphilis will result in an adverse outcome.^5^ Missed infections in pregnant women such as syphilis, HIV, and malaria and conditions such as hypertension, diabetes, and pre-eclampsia are also important preventable risk factors for stillbirths.^4^ Adequate antenatal care is also becoming increasingly important as the burden of non-communicable diseases grows and more women present to clinics with chronic diseases.^6^ In addition to its direct effect on health, higher quality antenatal care has also been linked to a higher likelihood of retention in care and of giving birth in a health facility, which might further improve maternal and newborn outcomes.^7^, ^8^, ^9^

The Sustainable Development Goals call for an equitable distribution of health gains.^10^ However, to date most of the work illuminating inequities in health service delivery has focused on access to care. Numerous studies have shown that certain population groups are consistently less likely to have access to and to use health services.^11^, ^12^ These studies have revealed systematic pro-rich inequalities for virtually all coverage indicators.^1^ Nonetheless, differences in the quality of care received by disadvantaged people have not been systematically examined. Some studies have shown equitable levels of low-quality care, regardless of poverty status, whereas others have revealed educational and wealth gradients in quality.^13^, ^14^, ^15^, ^16^

We aimed to address this gap by describing inequalities in antenatal care quality across the largest possible set of countries using comparable indicators and a standardised measurement approach. We described the magnitude of between-country and within-country inequalities in antenatal care quality and assessed the extent to which these inequalities could be explained by other geographical and sociodemographic factors.

**Research in context**

**Evidence before this study**

Many studies have described socioeconomic inequalities in access to health services and show that poorer people across low-income and middle-income countries (LMICs) are less likely to use health services than are wealthier people. Emerging data now also show that the quality of the care accessed in LMICs is often low. However, whether poorer people in these regions consistently receive poorer quality care remains unclear. We searched PubMed with the terms “maternal health”, “quality”, and “equity” to identify articles published in English between Jan 1, 2007, and Mar 1, 2018, and identified few publications addressing socioeconomic inequalities in maternal health-care quality. Some studies have shown equitable levels of poor-quality care whereas others have found evidence of a wealth and educational gradient in the quality of services. One study analysed socioeconomic differences in the quality of antenatal services across 59 countries. However, this study did not provide country-specific results nor did it consider differences in measurement of quality across countries.

**Added value of this study**

Using comparable indicators and a standardised measurement approach, our study quantified the magnitude of inequalities in antenatal care quality and compared results across 91 LMICs from three different income groups. Despite high antenatal care coverage, we found that nearly a third of women did not have their blood pressure checked and their urine and blood tested at any point during their pregnancy. On average, the wealthiest women were four times more likely to report these three services during antenatal care than were the poorest women. These inequities were largest in low-income countries, where the wealthiest women were nearly ten times more likely to report good quality care.

**Implications of all the available evidence**

Many countries that have reached high levels of antenatal care coverage have much lower and inequitable levels of quality. We found that the poorest women in the poorest countries receive substantially lower quality care during pregnancy. Implications of this study are relevant to the measurement and improvement of health service quality. Current evidence highlights a clear need to move from measurement of coverage alone to measurement of effective, quality-corrected coverage and equity. Available evidence also points to the importance that quality improvement efforts begin in areas with the greatest quality gaps and explicitly consider poor and vulnerable populations to ensure that no one is left behind.

## Methods

### Data sources

We selected all Demographic and Health Surveys (DHS) and Multiple Indicator Cluster Surveys (MICS) done in LMICs in the past 10 years and included the most recent survey for each country (as of Jan 15, 2018). The DHS and MICS, respectively funded by the US Agency for International Development and the United Nations Children's Fund, gather data on a range of population health indicators with a strong focus on maternal and child health. Standardised questionnaires ensure that data collected are comparable across countries. Sampling strategies and methodology have been described previously.^17^, ^18^ Our population of interest included all women of reproductive age (15–49 years) who had at least one livebirth in the past 2 years (MICS) or 5 years (DHS).

### Coverage and quality measures

In each country, we estimated antenatal care coverage as the proportion of women who had at least one antenatal care visit with a skilled provider during their last pregnancy. We used country-specific definitions of skilled providers as defined in the DHS and MICS. These included doctors, nurses, midwives, and country-specific skilled providers (such as maternal and child health aides in Sierra Leone and health extension workers in Ethiopia). Country-specific definitions of skilled antenatal care providers are available in the appendix.

Guided by the framework of the *Lancet Global Health* Commission on High-Quality Health Systems in the SDG Era^2^ and by the WHO recommendations^3^ on antenatal care for a positive pregnancy experience, we assessed the availability of potential indicators of antenatal care quality in household surveys. Quality antenatal care involves the provision of respectful, evidence-based care including appropriate patient assessments such as history questions, examinations, and diagnostic tests (eg, full blood count testing and urine culture); appropriate preventive and curative treatments (eg, tetanus toxoid vaccination and iron supplementation); and patient counselling and education (eg, on healthy eating and signs of complications).

In the DHS and MICS, women who reported attending antenatal care were asked whether they received specific services during consultations. We found 13 indicators related to antenatal care quality: weight and height measurement, blood pressure monitoring, urine and blood samples taken, HIV testing and counselling, tetanus vaccination, iron supplements, malaria prophylaxis, drugs for intestinal worms, counselling on signs of complications, and counselling on where to go in case of complications.

The number of items collected varied across countries and only three indicators remained consistently measured in all countries and relevant in all contexts: blood pressure monitoring and urine and blood testing. To obtain a comparable measure across the largest possible set of countries, we limited our estimate of antenatal care quality to these three indicators. Antenatal care quality was therefore included as a binary outcome measuring the proportion of women who reported having their blood pressure checked and giving a urine and blood sample at any point during pregnancy among those who sought care from skilled providers.

These three services do not comprise the full range of necessary antenatal care services and offer a limited view of antenatal care quality. However, they are recommended by WHO as essential components of antenatal care and are crucial to the detection of several pregnancy risks including hypertension, pre-eclampsia, infections, anaemia, and nutritional deficiencies.^3^ Our quality measure is also limited by the fact that information on the specific urine and blood tests done is not available in the DHS and MICS; women were asked whether they gave urine and blood specimens but not what the tests were for.

As a sensitivity analysis, and given the importance of counselling for the detection of pregnancy complications, we also estimated antenatal care quality by including a fourth indicator available in 55 (60%) of 91 countries. This indicator measured whether women were counselled on potential danger signs to look out for during pregnancy or where to go in case of a complication. The survey questions for these four indicators are shown in the appendix.

### Independent variables

At the country level, we included gross domestic product (GDP) per capita and country income groups specific to the survey year based on World Bank classification as independent variables. At the individual level, we used the wealth index constructed by the DHS and MICS as an estimate of socioeconomic position. The wealth index is based on a household's ownership of selected assets, housing construction materials, and types of water access and sanitation facilities and is estimated by principal component analysis. As a further independent variable at the individual level, we also used the woman's educational attainment based on country-specific categories. Most surveys contained a variable with six categories (no education, attended primary, completed primary, attended secondary, completed secondary, or attended higher education). A few surveys used between three and five education categories, making inequality measured by education groups less comparable across countries. We also used the woman's age at childbirth categorised into three groups (15–19 years, 20–35 years, and 35–49 years); her place of residence (urban or rural); region, state, or province of residence; and the total number of antenatal care visits attended (modelled as a continuous variable).

### Statistical analysis

To assess inequalities between countries, we ranked countries by levels of antenatal care coverage and quality and compared results across country income groups (low income, lower-middle income, and upper-middle income). We also plotted antenatal care coverage and quality against GDP per capita.

To assess inequalities within countries, we ranked women using the wealth index and assigned a relative ranking based on their position in the cumulative wealth distribution. We then measured inequalities in antenatal care coverage and quality using the slope index of inequality (SII) and the relative index of inequality (RII).^19^, ^20^ Inequalities have been found to differ substantially when measured according to dimensions of inequality other than wealth;^12^ therefore, we also measured the SII and RII using the woman's education and show these results in the appendix. The SII expresses the absolute percentage point difference in antenatal care coverage or quality between the predicted poorest and richest in the wealth distribution, assuming a linear relation between social rank and the outcome.^21^ The RII expresses the ratio of the predicted outcomes between the two extremes of the wealth distribution, assuming a log-linear relation.^21^ We used logistic regression models to estimate the association between the woman's relative rank and her antenatal care outcomes. The SII and RII were obtained using marginal effects and the *lincom* and *nlcom* post-estimation commands in Stata version 14.2. Individual-level sampling weights and robust SEs were used in all regressions.^17^, ^18^ To summarise coverage, quality, and inequalities across countries, we pooled the estimates using random-effects meta-analyses weighted by the inverse variance of the estimates.^22^ We assessed heterogeneity across countries using *I*^2^ statistics.

Finally, to assess the extent to which wealth-related inequalities in antenatal care quality could be explained by other geographical and socioeconomic determinants, we sequentially added five variables to the wealth rank in the regression models used to estimate the SII and RII: the woman's education and age group, urban versus rural residence, subnational region, and total number of antenatal care visits (because women who attend more visits might have more opportunities to receive the three services we assessed).

### Role of the funding source

The study sponsors did not have any role in study design, data analysis, data interpretation, writing of the report, or submission for publication. The corresponding author had full access to all the data in the study and had final responsibility for the decision to submit for publication.

## Results

**Figure 1:**
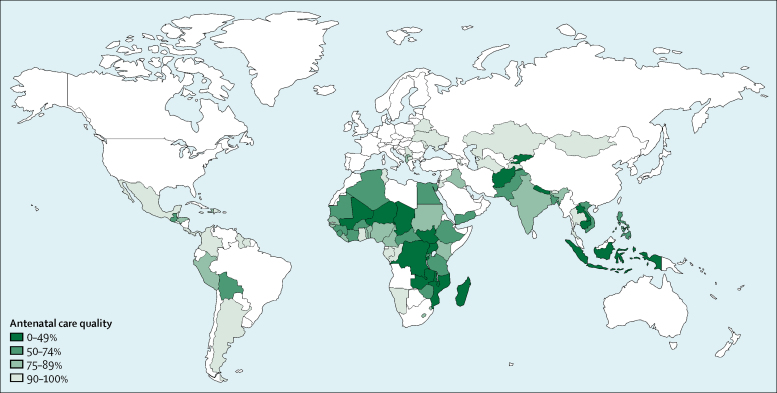
Antenatal care quality in 91 low-income and middle-income countries Non-coloured regions had no data available or were not relevant to this analysis. Antenatal care quality is defined as the proportion of women who report blood pressure monitoring and urine and blood testing at any point during the pregnancy among those who had at least one visit with a skilled provider.

**Figure 2:**
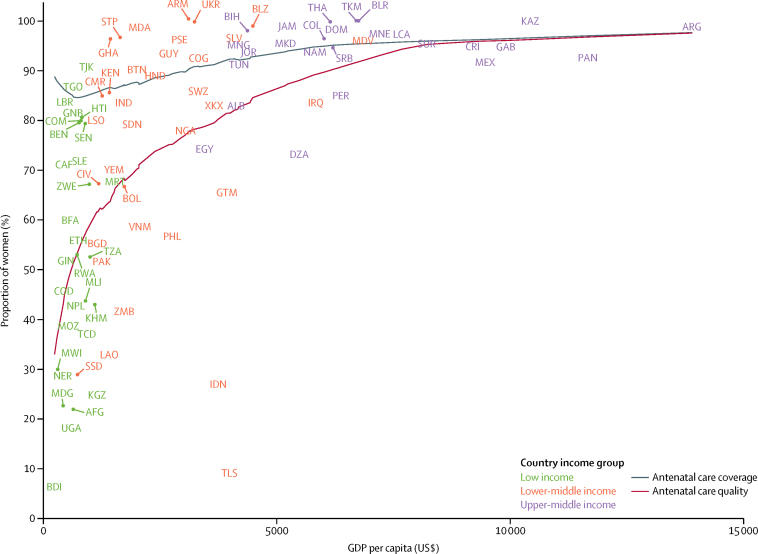
Antenatal care quality and coverage by GDP per capita in 91 low-income and middle-income countries Countries are represented with International Organization for Standardization country codes. Antenatal care quality is defined as the proportion of women who report blood pressure monitoring and urine and blood testing at any point during the pregnancy among those who had at least one visit with a skilled antenatal care provider. Antenatal care coverage is defined as the proportion of women with at least one livebirth in the past 2 or 5 years who had at least one visit with a skilled provider. GDP=gross domestic product.

We included surveys from 30 low-income countries, 35 lower-middle-income countries, and 26 upper-middle-income countries, resulting in an analytical sample of 671 697 women in 91 LMICs. The included surveys were done over a period of 9 years (2007–16), with 84 (92%) of them taking place between 2010 and 2016. Antenatal care coverage was high: on average, 89·7% (95% CI 88·0–91·4) of women attended at least one antenatal care visit with a skilled provider (table 1; appendix). However, only 72·9% (69·1–76·8) of these women reported getting their blood pressure checked and their urine and blood taken at any point during their pregnancy (table 1), ranging from only 6% in Burundi to nearly 100% in Armenia, Belarus, Kazakhstan, and Ukraine (figure 1; appendix). We found that quality lagged behind coverage the most in low-income countries, where 86·6% (83·4–89·7) of women accessed care but only 53·8% (44·3–63·3) on average reported receiving the three services (table 1). Similarly, in lower-middle-income countries, coverage was 87·8% (84·4–91·2) and quality 74·8% (68·6–80·9). In upper-middle-income countries, levels of coverage (96·1%, 95% CI 95·2–97·0) and quality (93·3%, 91·4–95·2) were closer together. When considering associations between national levels of antenatal care coverage, antenatal care quality, and GDP per capita, we found that quality was more strongly correlated with a country's GDP per capita than coverage (figure 2; Spearman's ρ 0·71).

**Table 1:** Antenatal care coverage, quality, and inequalities in 91 low-income and middle-income countries by income group

		**Global (n=91)**	**Low income (n=30)**	**Lower-middle income (n=35)**	**Upper-middle income (n=26)**
**Coverage**					
Antenatal care coverage*		89·7% (88·0–91·4)	86·6% (83·4–89·7)	87·8% (84·4–91·2)	96·1% (95·2–97·0)
**Quality**					
Antenatal care quality†		72·9% (69·1–76·8)	53·8% (44·3–63·3)	74·8% (68·6–80·9)	93·3% (91·4–95·2)
	Blood pressure measured	91·7% (90·7–92·7)	84·9% (81·7–88·2)	93·3% (91·9–94·8)	98·2% (97·8–98·7)
	Blood sample taken	82·0% (79·6–84·4)	71·8% (64·6–79·0)	81·1% (77·2–85·0)	96·0% (94·7–97·3)
	Urine sample taken	77·9% (75·0–80·9)	62·3% (52·6–71·9)	80·4% (76·9–84·0)	94·6% (92·8–96·4)
**Inequalities in antenatal care quality**‡					
SII		0·27 (0·23–0·30)	0·38 (0·31–0·44)	0·30 (0·21–0·39)	0·09 (0·06–0·12)
RII		4·01 (3·90–4·13)	9·63 (8·10–11·45)	5·30 (4·83–5·81)	3·01 (2·93–3·10)

Data are mean (95% CI). Estimates are pooled across countries and income groups using inverse-variance-weighted random-effects meta-analysis. *I*^2^ >90% in all analyses. SII=slope index of inequality. RII=relative index of inequality.

* Antenatal care coverage is defined as the proportion of women with at least one livebirth in the past 2 or 5 years who had at least one visit with a skilled provider.

† Antenatal care quality is defined as the proportion of women who report blood pressure monitoring and urine and blood testing at any point during the pregnancy among those who had at least one visit with a skilled provider.

‡ An SII value of 0·27 indicates that the proportion of women who report good quality care is 27 percentage points higher on average at the top of the wealth distribution compared with the bottom. An RII value of 4·01 indicates that the wealthiest women are on average four times more likely to report god quality care than the poorest.

**Figure 3:**
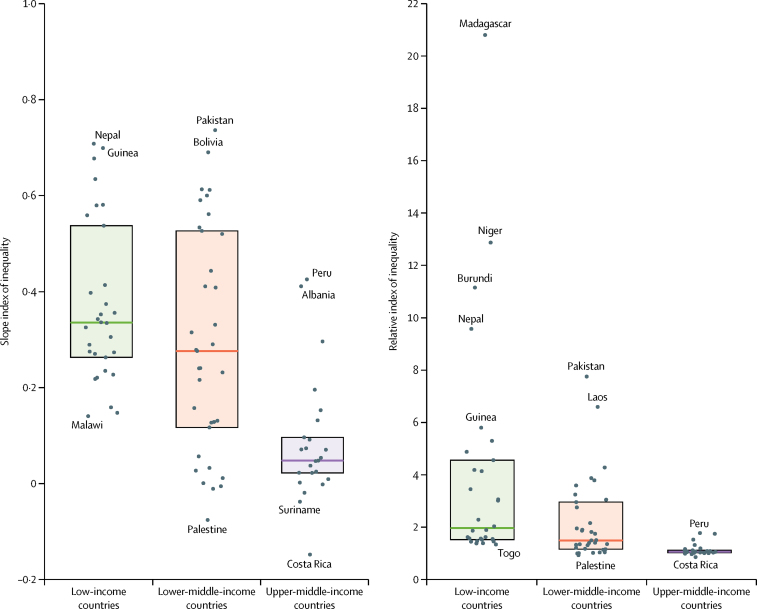
Slope and relative indices of inequality in antenatal care quality by country income group Dots represent country-specific point estimates of the slope and relative indices of inequality in antenatal care quality. Countries at the extremes of the scales are named. Shaded boxes represent the IQR of inequality across countries and horizontal lines delineate the median. Country-specific estimates and confidence intervals are shown in the appendix.

We found significant inequalities in antenatal care quality, both absolute and relative, in 70 (78%) of the 91 countries (appendix). On average across all countries, the wealthiest women were four times more likely to report the three services considered than were the poorest women in their same country (table 1). Inequalities tended to be larger in low-income countries, where the wealthiest women were nearly ten times more likely to receive good quality care than were the poorest (table 1). On the absolute scale, the pooled SII reflected that, on average in any given country, the proportion of women who report the three services is 27 percentage points higher at the top of the socioeconomic distribution compared with the bottom (table 1). Relative and absolute inequalities were largest in Madagascar (RII 20·8, 95% CI 14·9–26·7) and Pakistan (SII 0·74, 0·70–0·77), respectively (figure 3). Inequalities according to maternal education were similar, on average, to those according to the wealth index (appendix).

**Figure 4:**
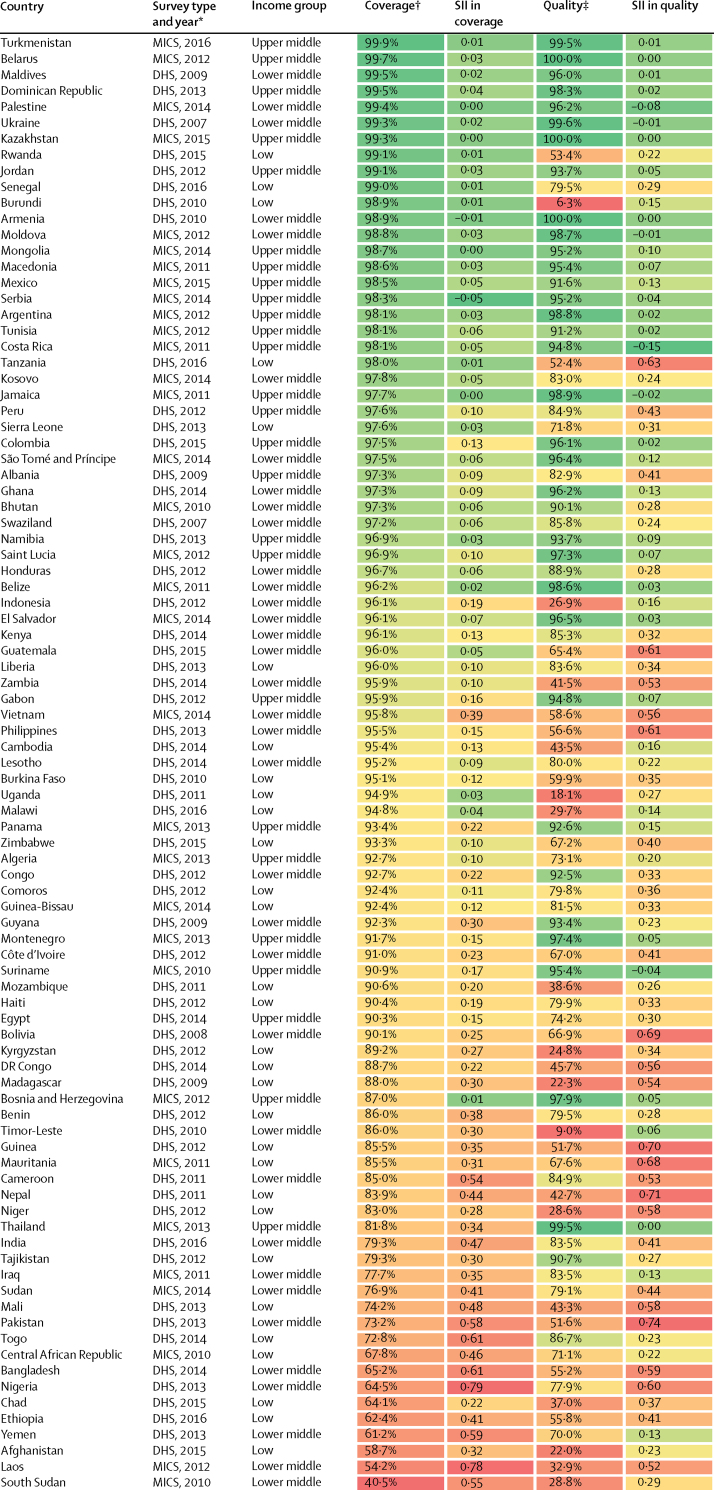
Antenatal care coverage, quality, and equity in 91 low-income and middle-income countries Countries are ranked by level of antenatal care coverage. Dark green indicates high coverage and equity (ie, smaller inequalities) and red indicates low coverage and equity (ie, greater inequalities). DHS=Demographic and Health Surveys. MICS=Multiple Indicator Cluster Surveys. SII=slope index of inequality. *Recall period is limited to 2 years in MICS and to 5 years in DHS. †Antenatal care coverage is defined as the proportion of women with at least one livebirth in the past 2 or 5 years who had at least one visit with a skilled provider. ‡Antenatal care quality is defined as the proportion of women who report blood pressure monitoring and urine and blood testing at any point during the pregnancy among those who had at least one visit with a skilled antenatal care provider.

Many countries with high levels of coverage had low and inequitable levels of quality (figure 4). For example, in Rwanda, Burundi, Tanzania, Indonesia, Zambia, Vietnam, Philippines, Cambodia, Burkina Faso, Uganda, Malawi, and Mozambique, more than 90% of women accessed skilled antenatal care but less than 60% reported the three services (figure 4). In some countries, quality was low across all wealth groups. For example, in Timor-Leste, Burundi, and Afghanistan, less than 30% of women in the richest wealth quintile reported the three services (appendix). We found even lower levels of quality when including a fourth indicator (counselling on complications). Across the 55 countries with available data, only 44% of women reported receiving the four services during antenatal care (appendix).

Adjustment for subnational regions and urban residence explained an important proportion of absolute wealth-related inequalities in antenatal care quality (table 2). The woman's education and number of antenatal care visits explained a similar proportion of both absolute and relative inequalities. Adjustment for maternal age had no effect on the magnitude of inequalities. In addition, important inequalities remained after adjustment for all five covariates (table 2).

**Table 2:** Inequalities in antenatal care quality in 91 low-income and middle-income countries adjusted for geographical and sociodemographic factors

	**SII in antenatal care quality**	**RII in antenatal care quality**
Crude	0·27 (0·23–0·30)	4·01 (3·90–4·13)
Adjusted for region, state, or province	0·22 (0·18–0·26)	4·12 (3·93–4·31)
Adjusted for urban residence	0·21 (0·17–0·25)	3·99 (3·81–4·17)
Adjusted for age group*	0·26 (0·22–0·31)	4·45 (4·26–4·64)
Adjusted for education group†	0·22 (0·19–0·25)	3·73 (3·62–3·85)
Adjusted for number of antenatal care visits‡	0·22 (0·19–0·24)	3·55 (3·48–3·63)
Adjusted for all five covariates	0·11 (0·09–0·13)	3·20 (3·11–3·30)

Data are estimate (95% CI). Antenatal care quality is defined as the proportion of women who report blood pressure monitoring and urine and blood testing at any point during pregnancy among those who had at least one visit with a skilled ANC provider. Estimates are pooled across countries and income groups using inverse-variance-weighted random-effects meta-analysis. *I*^2^ >90% in all analyses. SII=slope index of inequality. RII=relative index of inequality. DHS=Demographic and Health Surveys. MICS=Multiple Cluster Indicator Surveys.

* Age groups are 15–19 years, 20–35 years, and 35–49 years.

† Categories of educational attainment are country-specific categories available in the DHS and MICS.

‡ Number of antenatal care visits are modelled as continuous from one to 20.

## Discussion

This study provides a systematic analysis of inequalities in antenatal care quality based on the most recent nationally representative household surveys in 91 LMICs. Despite high antenatal care coverage across all country income groups, we found that nearly a third of women who accessed antenatal care did not receive a basic package of three services during their pregnancy. The correlation between antenatal care quality and GDP per capita was strong: in upper-middle-income countries, the proportion of women reporting the three services was 40 percentage points higher than in low-income countries. Within countries, the wealthiest women were four times more likely to report good quality antenatal care than the poorest.

The relevance of these findings for population health is highlighted by the dissonance between coverage figures and maternal and newborn survival. Globally, 86% of pregnant women now access antenatal care with skilled providers at least once during pregnancy and 78% deliver with skilled birth attendants.^23^ Yet, every year, an estimated 2·6 million babies are stillborn, 2·7 million newborns die in the first month of life, and 303 000 women die from preventable causes related to childbirth and pregnancy, 93% of whom live in low-income and lower-middle-income countries.^24^ The new WHO antenatal care model recommends that women attend a minimum of eight antenatal care visits.^3^ However, with poor levels of quality, merely increasing the number of visits is unlikely to produce the desired health outcomes.

We found that poorer women receive substantially lower quality care. Adjusting for rurality, subnational region, age, education, and number of visits reduced the magnitude of SII by more than a half and of the RII by nearly a third. However, substantial socioeconomic inequality remained after adjustments for these covariates. Geographical location appears to explain a portion of the wealth gradient in antenatal care quality. Poorer people predominantly live in rural areas and regions with poorly functioning health systems. However, these broad geographical categories probably conceal important variation in quality of care.

Beyond the variables available from the DHS and MICS, other factors might explain why poor women get worse care. These factors could include availability of good facilities nearby, cost of diagnostic procedures, provider discrimination or bias, and women's empowerment and degree of patient activation to seek high-quality care.

Studies have shown that poorer women tend to access care locally or at nearby facilities and tend to live next to facilities with poorer structural quality.^14^, ^25^ These facilities might not have the basic amenities and equipment needed to provide the three services analysed in our study. Blood pressure cuffs, supplies to collect urine and blood specimens, and either on-site diagnostic capacity or a nearby laboratory are needed to provide antenatal care. However, in many health facilities even simple tests are often unavailable. Across ten countries, only 2% of facilities surveyed between 2007 and 2015 had eight diagnostic tests defined as essential for basic service readiness by the WHO, including those necessary for antenatal care, such as urine dipsticks for protein and glucose, syphilis rapid diagnostic tests, and HIV diagnostic capacity.^26^ Unfortunately, DHS and MICS do not easily permit linkage with facility surveys, so we were unable to assess the structural quality of the facilities used by the women in our study. Emerging data show that most LMICs do not have adequate infrastructure to support laboratory services.^27^ Without structural readiness, high-quality care is unlikely. However, a recent study across eight LMICs showed that the availability of equipment and supplies did not guarantee good quality care.^28^ Even some providers in well equipped facilities were found to provide poor care, and vice versa. Weaker facilities might also employ providers with lower clinical skills. Although we restricted the analysis to antenatal care received from skilled providers, definitions of skilled care vary across countries (appendix). Some countries designate certain cadres as skilled attendants despite them not having the requisite skills.^29^, ^30^ Poorer women might be seeking care from less competent providers, who do not have the knowledge and technical skills to conduct adequate antenatal care.

In addition, although antenatal care consultations are free in many countries, women are often charged for specific services or consumables. Studies from LMICs have reported important direct costs for antenatal care including for booking appointments, medicines, laboratory tests, ultrasound scans and donations.^31^, ^32^, ^33^ Out-of-pocket payments continue to be an important barrier in accessing care in LMICs. Unlike curative services, the benefits of preventive care such as antenatal care tend to be deferred rather than immediate and women might be less likely to pay for such services upfront, leaving them at higher risk of financial shocks in the case of an obstetric complication. Financial protection mechanisms are needed to ensure equitable access to high-quality antenatal care that includes all necessary laboratory tests. We found that blood pressure monitoring was more common than were blood and urine tests. Payments charged for diagnostic tests could explain this finding. Similar to another study,^34^ we also found that blood tests were more common than were urine tests, possibly reflecting a stronger commitment to HIV testing in many countries.

Discriminatory treatment from health workers could also explain why poorer women get worse care than their richer counterparts. Qualitative studies have revealed instances of differential treatment among disadvantaged women during childbirth, including inadequate care, insufficient attention from staff, and disrespectful or abusive care.^35^, ^36^ However, due to their poverty, low educational attainment, illiteracy, or ethnic background, these women might also be differentially treated during antenatal care, and might not be offered the same quality of care as wealthier women.

Finally, disadvantaged women might also lack the agency to advocate for themselves and demand high-quality care. Lower agency and sense of empowerment diminish women's ability to insist on higher quality services. Similarly, poor health literacy and knowledge of patients' rights and entitlements mean that certain women are unable to recognise what constitutes good care and have lower expectations during visits. Nonetheless, more work is needed to understand the factors responsible for inequities in health-care quality.

The low overall performance and extent of inequality we found for even basic elements of clinical care is consistent with research from the *Lancet Global Health* Commission on High Quality Health Systems in the SDG Era,^2^ which described wide disparities in health-care quality across country income groups. Poorly functioning health systems characterised by long wait times, short consultations, and inadequate and sometimes disrespectful care hinder women's ability to obtain the full range of antenatal care services and the follow-up needed to achieve desirable health outcomes.

Others have also described the presence of a quality-coverage gap in maternal and newborn care, whereby women and caregivers with multiple contacts with the health system report low coverage of crucial elements of care.^37^, ^38^ Even among women with ideal care-seeking patterns during pregnancy (ie, those who begin antenatal care in the first trimester and attend at least four visits), the content and quality of care provided remained inadequate.^34^

Our results are also consistent with studies on socioeconomic inequities in antenatal care quality conducted in Brazil, India, Kenya, and Mexico.^7^, ^14^, ^16^, ^39^ One study^40^ used data from 59 DHS and found evidence of a wealth and educational gradient in the content of antenatal care. However, this study did not provide country-specific results nor did it consider differences in measurement of antenatal care content across countries. To our knowledge, ours is the first study to provide a systematic analysis of inequalities in maternal health-care quality across a large set of LMICs using comparable indicators and a standardised measurement approach. We used the most recent, nationally representative household surveys, to obtain comparable estimates of inequality across 91 countries, and measured inequalities using the SII and RII. Unlike simple comparisons between the richest and poorest wealth quintiles, the SII and RII consider information on all subgroups in the population and offer more reliable international comparisons and a more complete representation of the shape of inequality.^19^, ^20^ We also presented both absolute and relative measures of inequalities because these can lead to different conclusions about the magnitude and trends in inequalities.^20^

Nonetheless, our study has several limitations. First, because of poor comparability of quality items across countries and between DHS and MICS, our quality measure consists of only three indicators and should thus be seen as a starting point for assessment of quality rather than a definitive measure. The poor quality and substantial inequalities we observe are likely to be magnified with a more complete assessment. For example, we found substantially lower quality when adding a fourth indicator related to counselling in a subset of countries. Second, DHS and MICS data are based on self-reports and recall bias could affect the reported measures of the different services received during antenatal care. However, validation studies have found small-to-moderate levels of bias in self-reported coverage of maternal health indicators, and more invasive interventions such as blood tests appeared to be more accurately reported.^41^, ^42^ Finally, our description of inequalities in antenatal care quality is limited by the fact that surveys were done over a period of 9 years (2007–16). However, 84 of the 91 surveys took place between 2010 and 2016. Differences in recall periods between DHS (pregnancies in the past 5 years) and MICS (past 2 years) also limits comparability across countries as longer recall periods could introduce greater measurement error.

Access to effective care is a core component of the human right to health. Our study showed that the poorest women in the poorest countries tend to receive substantially lower quality care during pregnancy. Our study highlights a clear need for better measurement and systematic improvement in health-care quality. In the Sustainable Development Goals (SDG) era, measurement should pivot from coverage to effective coverage and equity. Effective coverage is defined as the fraction of potential health gain that is actually delivered to the population through the health system and combines measurement of health service need, use, and quality.^43^ Equity in effective coverage should thus be used as the new metric to monitor progress towards universal health coverage.

Our study also points to the importance of quality improvement efforts to begin in areas with greatest quality gaps and explicitly consider the needs and experience of poor and vulnerable populations. Progressive universalism, an approach to universal health coverage, has shown to be successful at improving health equity.^44^ However, although identification of poor population groups is possible, implementing interventions that focus on these vulnerable groups every step of the way is challenging.^45^ As described above, a myriad of factors could be responsible for existing socioeconomic inequalities in health-care quality and the solutions will vary depending on the reasons why such inequalities arise. Additionally, even with pro-poor approaches, gradients might persist, particularly in countries with large baseline inequalities. In the SDG era, achieving parity in health outcomes between rich people and poor people, within and across countries, will require greater focus on the quality of health services and its equitable distribution.
