# Supplementary material for: Equity in antenatal care quality: an analysis of 91 national household surveys
Source: Lancet Glob Health. 2018 Oct 12;6(11):e1186–95. doi: 10.1016/S2214-109X(18)30389-9 (PMC6187112; doi:10.1016/S2214-109X(18)30389-9)

THE LANCET
Global Health

## Supplementary appendix

This appendix formed part of the original submission and has been peer reviewed. We post it as supplied by the authors.

Supplement to: Arsenault C, Jordan K, Lee D, et al. Equity in antenatal care quality: an analysis of 91 national household surveys. *Lancet Glob Health* 2018; **6:** e1186–95.

# Equity in antenatal care quality: an analysis of 91 national household surveys

## Supplementary material

## Appendix 1. Definitions of skilled antenatal care providers by country

| Country | Survey type | Year | Skilled antenatal care providers |
|---|---|---|---|
| Afghanistan | DHS | 2015 | Doctors, nurses/midwives, and auxiliary nurse/midwives |
| Albania | DHS | 2009 | Obstetrician/gynecologist, family doctor, and nurse, midwife. |
| Algeria | MICS | 2013 | Médecin, Infirmière / Sage-femme |
| Argentina | MICS | 2012 | Médico, Enfermera, Técnica obstétrica / partera |
| Armenia | DHS | 2010 | Doctor, nurse, midwife, and feldsher. |
| Bangladesh | DHS | 2014 | Qualified doctor, nurse/midwife/paramedic, FWV, CSBA, and SACMO |
| Belarus | MICS | 2012 | Doctors, nurse/midwives, medical assistants |
| Belize | MICS | 2011 | Doctor, Nurse / Midwife, Auxiliary midwife |
| Benin | DHS | 2012 | Doctors, nurses, Midwives, Nursing Assistants and Midwife Assistants. |
| Bhutan | MICS | 2010 | Doctor, Nurse / Midwife, HA/BHW, Asst. Clinical Officer (ACO) |
| Bolivia | DHS | 2008 | Doctor, nurse, auxiliary nurse |
| Bosnia Herzegovina | MICS | 2012 | Doctor, Nurse / Midwife |
| Burkina Faso | DHS | 2010 | Doctors, nurses, midwives, midwives, and trained midwives / birth attendants. |
| Burundi | DHS | 2010 | Physicians, nurses and midwives are considered health providers. |
| Cambodia | DHS | 2014 | Doctor, nurse, and midwife |
| Cameroon | DHS | 2011 | Doctors, nurses, midwives and nurses |
| Central African Rep | MICS | 2010 | Médecin, Infirmière / Sage-femme, Sage-femme auxiliaire |
| Chad | DHS | 2015 | Doctor, Nurse / Midwife, Matron and Hospital / Health Center Officer (this is "trained birth attendant") |
| Colombia | DHS | 2015 | Doctor, Nurse |
| Comoros | DHS | 2012 | Doctors, nurses and midwives. |
| Congo | DHS | 2012 | Doctors, nurses and midwives. |
| Costa Rica | MICS | 2011 | Médico general/obstetra, Enfermera general, Comadrona auxiliar, Enfermera obstétrica |
| Cote d'Ivoire | DHS | 2012 | Doctors, nurses, midwives and midwives / nursing assistants. |
| Dem. Republic of the Congo | DHS | 2014 | Trained staff including doctors, nurses and birth attendants |
| Dominican Republic | DHS | 2013 | Gynecologist / obstetrician, general practitioner, physician of another specialty, physician with unknown specialty and nurse. |
| Egypt | DHS | 2014 | Doctor or nurse/midwife. |
| El Salvador | MICS | 2014 | Médico, enfermera, enfermera auxiliar |
| Ethiopia | DHS | 2016 | Doctors and nurses/midwives, health officers, and health extension workers |
| Gabon | DHS | 2012 | Physicians, midwives, nurses, and nurse assistants are considered here as trained providers for antenatal care. |
| Ghana | DHS | 2014 | Doctor, nurse/midwife, and community health officer/nurse |
| Guatemala | DHS | 2015 | Doctor or nurse. |
| Guinea | DHS | 2012 | Doctors, nurses, midwives and midwives / nursing assistants. |
| Guinea Bissau | MICS | 2014 | Médico Enfermeira/parteira |
| Guyana | DHS | 2009 | Doctor, nurse, midwife, auxiliary nurse/midwife, and medex. |
| Haiti | DHS | 2012 | Physicians, nurses, midwifery nurses and nurse assistants are considered here as trained providers. |
| Honduras | DHS | 2012 | Doctor, nurse and auxiliary nurse |
| India | DHS | 2016 | Doctor, anm/nurse/mid-wife/lhv |
| Indonesia | DHS | 2012 | Doctor, obstetrician, nurse, midwife, and village midwife. |
| Iraq | MICS | 2011 | Doctor public Doctor private Nurse / Midwife |
| Jamaica | MICS | 2011 | Doctor Nurse / Midwife Auxiliary midwife |

| | | | |
|---|---|---|---|
| Jordan | DHS | 2012 | Doctor, nurse, and midwife |
| Kazakhstan | MICS | 2015 | Doctor Nurse / Midwife Feldsher |
| Kenya | DHS | 2014 | Doctor, nurse, and midwife |
| Kosovo | MICS | 2014 | Doctor Nurse / Midwife Auxiliary midwife |
| Kyrgyzstan | DHS | 2012 | Doctor, nurse, and midwife and feldsher. |
| Laos | MICS | 2012 | Doctor Nurse / Midwife Auxiliary midwife |
| Lesotho | DHS | 2014 | Doctor, nurse, and midwife |
| Liberia | DHS | 2013 | Doctor, nurse, and midwife and physician's assistant |
| Macedonia | MICS | 2011 | Doctor Midwife Obstetrical nurse |
| Madagascar | DHS | 2009 | Doctor, nurse, medical assistant |
| Malawi | DHS | 2016 | Doctors, clinical officers, medical assistants, nurses, and midwives. |
| Maldives | DHS | 2009 | Gynecologist, doctor, nurse, midwife, and community/family health worker |
| Mali | DHS | 2013 | Doctors, nurses, midwives and matrons. |
| Mauritania | MICS | 2011 | Médecin, Sage-femme, Infirmière, Accoucheuse auxiliaire |
| Mexico | MICS | 2015 | Médico, Enfermera, Partera profesional técnica |
| Moldova | MICS | 2012 | Doctor Nurse / Midwife |
| Mongolia | MICS | 2014 | Auxiliary midwife, Gynecologist Physician, Family/ Soum doctor Midwife Nurse |
| Montenegro | MICS | 2013 | Doctor Nurse / Midwife Auxiliary midwife |
| Mozambique | DHS | 2011 | Qualified health care agent includes physician, nurse, midwife, and ward / midwife assistant |
| Namibia | DHS | 2013 | Doctor, nurse, and midwife |
| Nepal | DHS | 2011 | Doctor, nurse, and midwife |
| Niger | DHS | 2012 | Doctor, nurse, and midwife |
| Nigeria | DHS | 2013 | Doctor, nurse, midwife, and auxiliary nurse/midwife. |
| Pakistan | DHS | 2013 | Doctor, nurse, midwife, and lady health visitor. |
| Palestine | MICS | 2014 | Doctor Nurse / Midwife |
| Panama | MICS | 2013 | Médico, Enfermera, Auxiliar de Enfermera, Comadrona, Curandero/Hierbero, Médico Tradicional Indígena |
| Peru | DHS | 2012 | Doctor, nurse, and midwife |
| Philippines | DHS | 2013 | Doctor, nurse, and midwife |
| Rwanda | DHS | 2015 | Doctor, nurse, medical assistant, and midwife. |
| Saint Lucia | MICS | 2012 | Doctor Nurse / Midwife |
| Sao Tome | MICS | 2014 | Doctor, nurse midwife, nurse, and nurse practitioner (auxiliary) |
| Senegal | DHS | 2016 | Doctor, nurse, and midwife |
| Serbia | MICS | 2014 | Doctor Nurse / Midwife |
| Sierra Leone | DHS | 2013 | Doctor, nurse, midwife, and MCH Aide |
| South Sudan | MICS | 2010 | Doctor, Midwife, Nurse / Midwife |
| Sudan | MICS | 2014 | Doctor Nurse / Midwife, Trained Midwife, Medical Assistant |
| Suriname | MICS | 2010 | Doctor Nurse / Midwife Auxiliary midwife |
| Swaziland | DHS | 2007 | Doctor, nurse/midwife/ auxiliary midwife |
| Tajikistan | DHS | 2012 | Doctor, nurse, midwife, and feldsher. |
| Tanzania | DHS | 2016 | Doctor/assistant medical officer (AMO), clinical officer, assistant clinical officer, nurse/midwife, assistant nurse, and MCH aide |
| Thailand | MICS | 2013 | Doctor Nurse/midwife |
| Timor Leste | DHS | 2010 | Doctor, nurse, midwife, and assistant nurse |
| Togo | DHS | 2014 | Doctors, medical assistants, nurses, midwives and auxiliary midwives |
| Tunisia | MICS | 2012 | Médecin Infirmière / Sage-femme, Sage-femme auxiliaire |
| Turkmenistan | MICS | 2016 | Doctor Nurse / Midwife Feldsher |

| | | | |
|---|---|---|---|
| Uganda | DHS | 2011 | Doctor, nurse/midwife, and medical assistant/clinical officer |
| Ukraine | DHS | 2007 | Doctor, nurse, and midwife |
| Vietnam | MICS | 2014 | Doctor Nurse / Midwife |
| Yemen | DHS | 2013 | Doctor, nurse, midwife, and auxiliary nurse/midwife |
| Zambia | DHS | 2014 | Doctor, clinical officer, and nurse/midwife |
| Zimbabwe | DHS | 2015 | Doctors, nurses and nurse/midwives |

**Appendix 2. Surveys questions on blood pressure monitoring, urine and blood testing and counseling on pregnancy complications during antenatal care**

**DHS 5 and 6**

- Did you see anyone for antenatal care for this pregnancy? (yes/no)
    - As part of your antenatal care during this pregnancy, were any of the following done at least once:
        - Was your blood pressure measured? (yes/no)
        - Did you give a urine sample? (yes/no)
        - Did you give a blood sample? (yes/no

**DHS 6**

- During any of your antenatal care visits, were you told about the signs of pregnancy complications? (yes/no)
- Were you told where to go if you had any of these complications? (yes/no)

**MICS**

- Did you see anyone for antenatal care during your pregnancy with (*NAME*)? (yes/no)
    - As part of your antenatal care during this pregnancy, were any of the following done at least once:
        - Was your blood pressure measured? (yes/no)
        - Did you give a urine sample? (yes/no)
        - Did you give a blood sample? (yes/no)

## Appendix 3. Antenatal care coverage and wealth-related inequalities in 91 low and middle-income countries

| Country | Antenatal care coverage | 95% CI | | Slope index of inequality in antenatal care coverage | 95% CI | | Relative index inequality in antenatal care coverage | 95% CI | |
|---|---|---|---|---|---|---|---|---|---|
| Afghanistan | 0.59 | 0.58 | 0.59 | 0.32 | 0.28 | 0.35 | 1.75 | 1.62 | 1.87 |
| Albania | 0.97 | 0.96 | 0.98 | 0.09 | 0.05 | 0.14 | 1.11 | 1.05 | 1.16 |
| Algeria | 0.93 | 0.92 | 0.93 | 0.10 | 0.07 | 0.13 | 1.12 | 1.08 | 1.15 |
| Argentina | 0.98 | 0.98 | 0.99 | 0.03 | 0.01 | 0.05 | 1.03 | 1.01 | 1.05 |
| Armenia | 0.99 | 0.98 | 0.99 | -0.01 | -0.02 | 0.01 | 0.99 | 0.98 | 1.01 |
| Bangladesh | 0.65 | 0.64 | 0.67 | 0.61 | 0.56 | 0.65 | 2.99 | 2.61 | 3.38 |
| Belarus | 1.00 | 0.99 | 1.00 | 0.03 | -0.03 | 0.10 | 1.04 | 0.96 | 1.11 |
| Belize | 0.96 | 0.95 | 0.98 | 0.02 | -0.03 | 0.07 | 1.02 | 0.97 | 1.07 |
| Benin | 0.86 | 0.85 | 0.87 | 0.38 | 0.35 | 0.40 | 1.62 | 1.55 | 1.69 |
| Bhutan | 0.97 | 0.97 | 0.98 | 0.06 | 0.03 | 0.09 | 1.06 | 1.03 | 1.10 |
| Bolivia | 0.90 | 0.89 | 0.91 | 0.25 | 0.22 | 0.29 | 1.35 | 1.28 | 1.41 |
| Bosnia Herzegovina | 0.87 | 0.84 | 0.89 | 0.01 | -0.11 | 0.13 | 1.01 | 0.87 | 1.15 |
| Burkina Faso | 0.95 | 0.95 | 0.96 | 0.12 | 0.10 | 0.14 | 1.13 | 1.11 | 1.16 |
| Burundi | 0.99 | 0.99 | 0.99 | 0.01 | -0.01 | 0.02 | 1.01 | 0.99 | 1.02 |
| Cambodia | 0.95 | 0.95 | 0.96 | 0.13 | 0.10 | 0.16 | 1.15 | 1.12 | 1.19 |
| Cameroon | 0.85 | 0.84 | 0.86 | 0.54 | 0.50 | 0.57 | 2.17 | 2.01 | 2.34 |
| Central African Rep | 0.68 | 0.66 | 0.69 | 0.46 | 0.41 | 0.51 | 2.05 | 1.86 | 2.25 |
| Chad | 0.64 | 0.63 | 0.65 | 0.22 | 0.18 | 0.25 | 1.41 | 1.33 | 1.49 |
| Colombia | 0.97 | 0.97 | 0.98 | 0.13 | 0.10 | 0.16 | 1.15 | 1.11 | 1.19 |
| Comoros | 0.92 | 0.91 | 0.94 | 0.11 | 0.06 | 0.16 | 1.13 | 1.06 | 1.20 |
| Congo | 0.93 | 0.92 | 0.93 | 0.22 | 0.19 | 0.25 | 1.29 | 1.24 | 1.33 |
| Costa Rica | 0.98 | 0.97 | 0.99 | 0.05 | 0.00 | 0.10 | 1.05 | 1.00 | 1.11 |
| Cote d'Ivoire | 0.91 | 0.90 | 0.92 | 0.23 | 0.20 | 0.27 | 1.31 | 1.25 | 1.38 |
| Dem. Republic of the Congo | 0.89 | 0.88 | 0.89 | 0.22 | 0.19 | 0.24 | 1.29 | 1.24 | 1.33 |
| Dominican Republic | 0.99 | 0.99 | 1.00 | 0.04 | 0.00 | 0.07 | 1.04 | 1.00 | 1.07 |
| Egypt | 0.90 | 0.90 | 0.91 | 0.15 | 0.13 | 0.17 | 1.18 | 1.15 | 1.22 |
| El Salvador | 0.96 | 0.95 | 0.97 | 0.07 | 0.05 | 0.10 | 1.08 | 1.05 | 1.11 |
| Ethiopia | 0.62 | 0.61 | 0.63 | 0.41 | 0.36 | 0.46 | 2.02 | 1.82 | 2.21 |
| Gabon | 0.96 | 0.95 | 0.97 | 0.16 | 0.12 | 0.21 | 1.20 | 1.14 | 1.26 |
| Ghana | 0.97 | 0.97 | 0.98 | 0.09 | 0.06 | 0.11 | 1.10 | 1.07 | 1.12 |
| Guatemala | 0.96 | 0.96 | 0.96 | 0.05 | 0.03 | 0.06 | 1.05 | 1.03 | 1.07 |
| Guinea | 0.86 | 0.85 | 0.86 | 0.35 | 0.31 | 0.38 | 1.55 | 1.46 | 1.64 |
| Guinea Bissau | 0.92 | 0.91 | 0.93 | 0.12 | 0.09 | 0.15 | 1.14 | 1.10 | 1.18 |
| Guyana | 0.92 | 0.91 | 0.94 | 0.30 | 0.23 | 0.37 | 1.43 | 1.28 | 1.58 |
| Haiti | 0.90 | 0.90 | 0.91 | 0.19 | 0.15 | 0.22 | 1.24 | 1.18 | 1.29 |
| Honduras | 0.97 | 0.96 | 0.97 | 0.06 | 0.05 | 0.08 | 1.07 | 1.05 | 1.09 |
| India | 0.79 | 0.79 | 0.80 | 0.47 | 0.46 | 0.48 | 1.95 | 1.92 | 1.98 |
| Indonesia | 0.96 | 0.96 | 0.96 | 0.19 | 0.17 | 0.22 | 1.24 | 1.20 | 1.28 |
| Iraq | 0.78 | 0.77 | 0.78 | 0.35 | 0.32 | 0.38 | 1.61 | 1.53 | 1.68 |
| Jamaica | 0.98 | 0.97 | 0.99 | 0.00 | -0.05 | 0.06 | 1.00 | 0.95 | 1.06 |
| Jordan | 0.99 | 0.99 | 0.99 | 0.03 | 0.01 | 0.05 | 1.03 | 1.01 | 1.05 |
| Kazakhstan | 0.99 | 0.99 | 1.00 | 0.00 | -0.01 | 0.02 | 1.00 | 0.99 | 1.02 |
| Kenya | 0.96 | 0.96 | 0.96 | 0.13 | 0.11 | 0.15 | 1.15 | 1.12 | 1.17 |
| Kosovo | 0.98 | 0.97 | 0.99 | 0.05 | 0.00 | 0.11 | 1.06 | 1.00 | 1.12 |
| Kyrgyzstan | 0.89 | 0.88 | 0.90 | 0.27 | 0.24 | 0.31 | 1.38 | 1.32 | 1.45 |
| Laos | 0.54 | 0.53 | 0.56 | 0.78 | 0.75 | 0.81 | 5.47 | 4.80 | 6.13 |
| Lesotho | 0.95 | 0.94 | 0.96 | 0.09 | 0.06 | 0.13 | 1.10 | 1.06 | 1.14 |
| Liberia | 0.96 | 0.95 | 0.97 | 0.10 | 0.08 | 0.13 | 1.11 | 1.08 | 1.14 |
| Macedonia | 0.99 | 0.98 | 1.00 | 0.03 | -0.01 | 0.07 | 1.03 | 0.99 | 1.08 |
| Madagascar | 0.88 | 0.87 | 0.89 | 0.30 | 0.27 | 0.33 | 1.45 | 1.39 | 1.51 |
| Malawi | 0.95 | 0.94 | 0.95 | 0.04 | 0.03 | 0.06 | 1.05 | 1.03 | 1.06 |

| | | | | | | | | | |
|---|---|---|---|---|---|---|---|---|---|
| Maldives | 1.00 | 0.99 | 1.00 | 0.02 | 0.01 | 0.04 | 1.03 | 1.01 | 1.04 |
| Mali | 0.74 | 0.73 | 0.75 | 0.48 | 0.44 | 0.51 | 2.05 | 1.91 | 2.19 |
| Mauritania | 0.85 | 0.84 | 0.87 | 0.31 | 0.26 | 0.35 | 1.46 | 1.37 | 1.54 |
| Mexico | 0.98 | 0.98 | 0.99 | 0.05 | 0.02 | 0.07 | 1.05 | 1.02 | 1.08 |
| Moldova | 0.99 | 0.98 | 1.00 | 0.03 | -0.02 | 0.08 | 1.03 | 0.98 | 1.08 |
| Mongolia | 0.99 | 0.98 | 0.99 | 0.00 | -0.02 | 0.01 | 1.00 | 0.98 | 1.01 |
| Montenegro | 0.92 | 0.89 | 0.94 | 0.15 | 0.05 | 0.26 | 1.18 | 1.04 | 1.33 |
| Mozambique | 0.91 | 0.90 | 0.91 | 0.20 | 0.17 | 0.23 | 1.26 | 1.21 | 1.31 |
| Namibia | 0.97 | 0.96 | 0.97 | 0.03 | 0.00 | 0.05 | 1.03 | 1.00 | 1.05 |
| Nepal | 0.84 | 0.83 | 0.85 | 0.44 | 0.40 | 0.48 | 1.82 | 1.67 | 1.96 |
| Niger | 0.83 | 0.82 | 0.84 | 0.28 | 0.25 | 0.32 | 1.43 | 1.36 | 1.50 |
| Nigeria | 0.65 | 0.64 | 0.65 | 0.79 | 0.77 | 0.80 | 5.44 | 5.08 | 5.79 |
| Pakistan | 0.73 | 0.72 | 0.74 | 0.58 | 0.54 | 0.61 | 2.54 | 2.29 | 2.78 |
| Palestine | 0.99 | 0.99 | 1.00 | 0.00 | -0.01 | 0.01 | 1.00 | 0.99 | 1.01 |
| Panama | 0.93 | 0.92 | 0.94 | 0.22 | 0.15 | 0.28 | 1.28 | 1.17 | 1.38 |
| Peru | 0.98 | 0.97 | 0.98 | 0.10 | 0.08 | 0.12 | 1.11 | 1.08 | 1.14 |
| Philippines | 0.96 | 0.95 | 0.96 | 0.15 | 0.12 | 0.18 | 1.18 | 1.14 | 1.22 |
| Rwanda | 0.99 | 0.99 | 0.99 | 0.01 | 0.00 | 0.02 | 1.01 | 1.00 | 1.02 |
| Saint Lucia | 0.97 | 0.93 | 1.00 | 0.10 | -0.09 | 0.29 | 1.11 | 0.88 | 1.34 |
| Sao Tome | 0.97 | 0.96 | 0.99 | 0.06 | 0.02 | 0.11 | 1.07 | 1.02 | 1.12 |
| Senegal | 0.99 | 0.99 | 0.99 | 0.01 | 0.00 | 0.02 | 1.01 | 1.00 | 1.02 |
| Serbia | 0.98 | 0.98 | 0.99 | -0.05 | -0.19 | 0.09 | 0.95 | 0.81 | 1.09 |
| Sierra Leone | 0.98 | 0.97 | 0.98 | 0.03 | 0.02 | 0.04 | 1.03 | 1.02 | 1.05 |
| South Sudan | 0.41 | 0.39 | 0.42 | 0.55 | 0.51 | 0.59 | 4.53 | 3.81 | 5.25 |
| Sudan | 0.77 | 0.76 | 0.78 | 0.41 | 0.37 | 0.46 | 1.78 | 1.65 | 1.90 |
| Suriname | 0.91 | 0.89 | 0.92 | 0.17 | 0.11 | 0.23 | 1.21 | 1.12 | 1.29 |
| Swaziland | 0.97 | 0.97 | 0.98 | 0.06 | 0.03 | 0.09 | 1.07 | 1.03 | 1.10 |
| Tajikistan | 0.79 | 0.78 | 0.81 | 0.30 | 0.25 | 0.35 | 1.48 | 1.37 | 1.60 |
| Tanzania | 0.98 | 0.98 | 0.98 | 0.01 | 0.00 | 0.03 | 1.01 | 1.00 | 1.03 |
| Thailand | 0.82 | 0.80 | 0.83 | 0.34 | 0.25 | 0.42 | 1.55 | 1.35 | 1.75 |
| Timor Leste | 0.86 | 0.85 | 0.87 | 0.30 | 0.27 | 0.33 | 1.45 | 1.38 | 1.52 |
| Togo | 0.73 | 0.72 | 0.74 | 0.61 | 0.58 | 0.64 | 2.79 | 2.56 | 3.01 |
| Tunisia | 0.98 | 0.97 | 0.99 | 0.06 | 0.02 | 0.09 | 1.06 | 1.02 | 1.10 |
| Turkmenistan | 1.00 | 1.00 | 1.00 | 0.01 | -0.01 | 0.02 | 1.01 | 0.99 | 1.02 |
| Uganda | 0.95 | 0.94 | 0.96 | 0.03 | 0.00 | 0.05 | 1.03 | 1.00 | 1.06 |
| Ukraine | 0.99 | 0.99 | 1.00 | 0.02 | -0.01 | 0.04 | 1.02 | 0.99 | 1.04 |
| Vietnam | 0.96 | 0.95 | 0.97 | 0.39 | 0.27 | 0.51 | 1.64 | 1.33 | 1.96 |
| Yemen | 0.61 | 0.60 | 0.62 | 0.59 | 0.55 | 0.62 | 3.02 | 2.77 | 3.27 |
| Zambia | 0.96 | 0.96 | 0.96 | 0.10 | 0.08 | 0.12 | 1.11 | 1.09 | 1.13 |
| Zimbabwe | 0.93 | 0.93 | 0.94 | 0.10 | 0.07 | 0.13 | 1.11 | 1.08 | 1.15 |

## Appendix 4. Antenatal care quality and wealth-related inequalities in 91 low and middle-income countries

| Country | Antenatal care quality | 95% CI | | Slope index of inequality in antenatal care quality | 95% CI | | Relative index of inequality in antenatal care quality | 95% CI | |
|---|---|---|---|---|---|---|---|---|---|
| Afghanistan | 0.22 | 0.21 | 0.23 | 0.23 | 0.18 | 0.27 | 3.06 | 2.41 | 3.70 |
| Albania | 0.83 | 0.81 | 0.85 | 0.41 | 0.33 | 0.49 | 1.74 | 1.49 | 1.99 |
| Algeria | 0.73 | 0.72 | 0.74 | 0.20 | 0.14 | 0.25 | 1.31 | 1.21 | 1.41 |
| Argentina | 0.99 | 0.98 | 0.99 | 0.02 | 0.01 | 0.04 | 1.02 | 1.01 | 1.04 |
| Armenia | 1.00 | 1.00 | 1.00 | 0.00 | 0.00 | 0.00 | 1.00 | 1.00 | 1.00 |
| Bangladesh | 0.55 | 0.53 | 0.57 | 0.59 | 0.53 | 0.65 | 3.87 | 3.08 | 4.66 |
| Belarus | 1.00 | 1.00 | 1.00 | 0.00 | 0.00 | 0.00 | 1.00 | 1.00 | 1.00 |
| Belize | 0.99 | 0.98 | 0.99 | 0.03 | -0.01 | 0.06 | 1.03 | 0.99 | 1.06 |
| Benin | 0.80 | 0.79 | 0.80 | 0.28 | 0.24 | 0.31 | 1.44 | 1.37 | 1.51 |
| Bhutan | 0.90 | 0.89 | 0.91 | 0.28 | 0.22 | 0.33 | 1.39 | 1.28 | 1.49 |
| Bolivia | 0.67 | 0.66 | 0.68 | 0.69 | 0.66 | 0.72 | 3.78 | 3.36 | 4.21 |
| Bosnia Herzegovina | 0.98 | 0.97 | 0.99 | 0.05 | -0.05 | 0.14 | 1.05 | 0.94 | 1.16 |
| Burkina Faso | 0.60 | 0.59 | 0.61 | 0.35 | 0.32 | 0.39 | 1.86 | 1.74 | 1.98 |
| Burundi | 0.06 | 0.06 | 0.07 | 0.15 | 0.11 | 0.18 | 11.15 | 4.51 | 17.79 |
| Cambodia | 0.43 | 0.42 | 0.45 | 0.16 | 0.10 | 0.21 | 1.45 | 1.26 | 1.64 |
| Cameroon | 0.85 | 0.84 | 0.86 | 0.53 | 0.48 | 0.57 | 2.15 | 1.96 | 2.34 |
| Central African Rep | 0.71 | 0.69 | 0.73 | 0.22 | 0.15 | 0.29 | 1.37 | 1.22 | 1.52 |
| Chad | 0.37 | 0.36 | 0.38 | 0.37 | 0.33 | 0.42 | 3.01 | 2.57 | 3.45 |
| Colombia | 0.96 | 0.96 | 0.97 | 0.02 | 0.00 | 0.05 | 1.03 | 1.00 | 1.05 |
| Comoros | 0.80 | 0.78 | 0.82 | 0.36 | 0.28 | 0.44 | 1.62 | 1.41 | 1.82 |
| Congo | 0.92 | 0.92 | 0.93 | 0.33 | 0.29 | 0.37 | 1.50 | 1.41 | 1.58 |
| Costa Rica | 0.95 | 0.93 | 0.96 | -0.15 | -0.41 | 0.12 | 0.85 | 0.58 | 1.12 |
| Cote d'Ivoire | 0.67 | 0.66 | 0.68 | 0.41 | 0.36 | 0.46 | 1.95 | 1.75 | 2.14 |
| Dem. Republic of the Congo | 0.46 | 0.45 | 0.47 | 0.56 | 0.52 | 0.59 | 4.14 | 3.62 | 4.65 |
| Dominican Republic | 0.98 | 0.98 | 0.99 | 0.02 | 0.00 | 0.05 | 1.02 | 1.00 | 1.05 |
| Egypt | 0.74 | 0.73 | 0.75 | 0.30 | 0.26 | 0.33 | 1.52 | 1.44 | 1.59 |
| El Salvador | 0.97 | 0.96 | 0.97 | 0.03 | 0.00 | 0.07 | 1.03 | 1.00 | 1.07 |
| Ethiopia | 0.56 | 0.54 | 0.57 | 0.41 | 0.35 | 0.48 | 2.28 | 1.94 | 2.62 |
| Gabon | 0.95 | 0.94 | 0.95 | 0.07 | 0.04 | 0.11 | 1.08 | 1.04 | 1.12 |
| Ghana | 0.96 | 0.96 | 0.97 | 0.13 | 0.10 | 0.16 | 1.15 | 1.11 | 1.19 |
| Guatemala | 0.65 | 0.64 | 0.66 | 0.61 | 0.59 | 0.64 | 3.04 | 2.81 | 3.28 |
| Guinea | 0.52 | 0.50 | 0.53 | 0.70 | 0.66 | 0.74 | 5.80 | 4.81 | 6.78 |
| Guinea Bissau | 0.81 | 0.80 | 0.83 | 0.33 | 0.28 | 0.37 | 1.52 | 1.42 | 1.62 |
| Guyana | 0.93 | 0.92 | 0.95 | 0.23 | 0.13 | 0.33 | 1.30 | 1.13 | 1.48 |
| Haiti | 0.80 | 0.79 | 0.81 | 0.33 | 0.29 | 0.38 | 1.57 | 1.46 | 1.67 |
| Honduras | 0.89 | 0.88 | 0.90 | 0.28 | 0.25 | 0.31 | 1.40 | 1.34 | 1.45 |
| India | 0.84 | 0.83 | 0.84 | 0.41 | 0.40 | 0.42 | 1.74 | 1.71 | 1.77 |
| Indonesia | 0.27 | 0.26 | 0.28 | 0.16 | 0.12 | 0.19 | 1.81 | 1.57 | 2.06 |
| Iraq | 0.83 | 0.83 | 0.84 | 0.13 | 0.09 | 0.16 | 1.17 | 1.12 | 1.22 |
| Jamaica | 0.99 | 0.98 | 1.00 | -0.02 | -0.06 | 0.02 | 0.98 | 0.94 | 1.02 |
| Jordan | 0.94 | 0.93 | 0.94 | 0.05 | 0.01 | 0.08 | 1.05 | 1.01 | 1.09 |
| Kazakhstan | 1.00 | 1.00 | 1.00 | 0.00 | 0.00 | 0.01 | 1.00 | 1.00 | 1.01 |
| Kenya | 0.85 | 0.84 | 0.86 | 0.32 | 0.28 | 0.35 | 1.49 | 1.41 | 1.56 |
| Kosovo | 0.83 | 0.80 | 0.86 | 0.24 | 0.14 | 0.34 | 1.35 | 1.17 | 1.52 |
| Kyrgyzstan | 0.25 | 0.24 | 0.26 | 0.34 | 0.30 | 0.39 | 4.55 | 3.46 | 5.65 |
| Laos | 0.33 | 0.31 | 0.35 | 0.52 | 0.45 | 0.59 | 6.59 | 4.47 | 8.71 |
| Lesotho | 0.80 | 0.78 | 0.82 | 0.22 | 0.15 | 0.28 | 1.32 | 1.21 | 1.43 |
| Liberia | 0.84 | 0.83 | 0.85 | 0.34 | 0.30 | 0.38 | 1.54 | 1.45 | 1.63 |
| Macedonia | 0.95 | 0.94 | 0.97 | 0.07 | 0.00 | 0.15 | 1.08 | 0.99 | 1.16 |
| Madagascar | 0.22 | 0.21 | 0.23 | 0.54 | 0.50 | 0.57 | 20.80 | 14.90 | 26.70 |
| Malawi | 0.30 | 0.29 | 0.30 | 0.14 | 0.10 | 0.18 | 1.61 | 1.41 | 1.81 |

| | | | | | | | | | |
|---|---|---|---|---|---|---|---|---|---|
| Maldives | 0.96 | 0.95 | 0.97 | 0.01 | -0.02 | 0.04 | 1.01 | 0.98 | 1.04 |
| Mali | 0.43 | 0.42 | 0.45 | 0.58 | 0.54 | 0.62 | 5.29 | 4.36 | 6.22 |
| Mauritania | 0.68 | 0.66 | 0.69 | 0.68 | 0.63 | 0.72 | 3.45 | 3.00 | 3.89 |
| Mexico | 0.92 | 0.91 | 0.93 | 0.13 | 0.09 | 0.18 | 1.16 | 1.09 | 1.22 |
| Moldova | 0.99 | 0.98 | 1.00 | -0.01 | -0.03 | 0.02 | 0.99 | 0.97 | 1.02 |
| Mongolia | 0.95 | 0.94 | 0.96 | 0.10 | 0.06 | 0.13 | 1.11 | 1.07 | 1.15 |
| Montenegro | 0.97 | 0.96 | 0.99 | 0.05 | -0.02 | 0.12 | 1.06 | 0.98 | 1.14 |
| Mozambique | 0.39 | 0.37 | 0.40 | 0.26 | 0.22 | 0.31 | 2.03 | 1.75 | 2.30 |
| Namibia | 0.94 | 0.93 | 0.94 | 0.09 | 0.06 | 0.13 | 1.10 | 1.06 | 1.15 |
| Nepal | 0.43 | 0.41 | 0.44 | 0.71 | 0.67 | 0.75 | 9.57 | 7.32 | 11.83 |
| Niger | 0.29 | 0.28 | 0.30 | 0.58 | 0.55 | 0.61 | 12.86 | 9.88 | 15.85 |
| Nigeria | 0.78 | 0.77 | 0.79 | 0.60 | 0.57 | 0.63 | 2.75 | 2.54 | 2.96 |
| Pakistan | 0.52 | 0.50 | 0.53 | 0.74 | 0.70 | 0.77 | 7.75 | 6.09 | 9.41 |
| Palestine | 0.96 | 0.96 | 0.97 | -0.08 | -0.11 | -0.04 | 0.92 | 0.89 | 0.95 |
| Panama | 0.93 | 0.91 | 0.94 | 0.15 | 0.08 | 0.22 | 1.18 | 1.09 | 1.28 |
| Peru | 0.85 | 0.84 | 0.86 | 0.43 | 0.39 | 0.46 | 1.77 | 1.66 | 1.87 |
| Philippines | 0.57 | 0.55 | 0.58 | 0.61 | 0.58 | 0.65 | 3.59 | 3.20 | 3.97 |
| Rwanda | 0.53 | 0.52 | 0.55 | 0.22 | 0.17 | 0.26 | 1.51 | 1.38 | 1.65 |
| Saint Lucia | 0.97 | 0.94 | 1.01 | 0.07 | -0.10 | 0.24 | 1.08 | 0.88 | 1.28 |
| Sao Tome | 0.96 | 0.95 | 0.98 | 0.12 | 0.06 | 0.18 | 1.13 | 1.05 | 1.21 |
| Senegal | 0.79 | 0.78 | 0.81 | 0.29 | 0.24 | 0.34 | 1.46 | 1.35 | 1.57 |
| Serbia | 0.95 | 0.94 | 0.97 | 0.04 | -0.07 | 0.14 | 1.04 | 0.92 | 1.16 |
| Sierra Leone | 0.72 | 0.71 | 0.73 | 0.31 | 0.27 | 0.34 | 1.56 | 1.47 | 1.65 |
| South Sudan | 0.29 | 0.27 | 0.31 | 0.29 | 0.22 | 0.36 | 3.24 | 2.07 | 4.41 |
| Sudan | 0.79 | 0.78 | 0.80 | 0.44 | 0.39 | 0.49 | 1.86 | 1.70 | 2.02 |
| Suriname | 0.95 | 0.94 | 0.97 | -0.04 | -0.09 | 0.01 | 0.96 | 0.91 | 1.01 |
| Swaziland | 0.86 | 0.84 | 0.87 | 0.24 | 0.18 | 0.30 | 1.34 | 1.24 | 1.44 |
| Tajikistan | 0.91 | 0.90 | 0.92 | 0.27 | 0.21 | 0.33 | 1.38 | 1.27 | 1.49 |
| Tanzania | 0.52 | 0.51 | 0.54 | 0.63 | 0.60 | 0.67 | 4.18 | 3.69 | 4.68 |
| Thailand | 0.99 | 0.99 | 1.00 | 0.00 | -0.02 | 0.01 | 1.00 | 0.98 | 1.01 |
| Timor Leste | 0.09 | 0.08 | 0.10 | 0.06 | 0.02 | 0.09 | 1.89 | 1.18 | 2.60 |
| Togo | 0.87 | 0.86 | 0.88 | 0.23 | 0.19 | 0.28 | 1.33 | 1.26 | 1.41 |
| Tunisia | 0.91 | 0.90 | 0.93 | 0.02 | -0.04 | 0.09 | 1.03 | 0.95 | 1.10 |
| Turkmenistan | 0.99 | 0.99 | 1.00 | 0.01 | -0.01 | 0.02 | 1.01 | 0.99 | 1.02 |
| Uganda | 0.18 | 0.17 | 0.19 | 0.27 | 0.22 | 0.32 | 4.87 | 3.39 | 6.36 |
| Ukraine | 1.00 | 0.99 | 1.00 | -0.01 | -0.03 | 0.01 | 0.99 | 0.97 | 1.01 |
| Vietnam | 0.59 | 0.56 | 0.61 | 0.56 | 0.48 | 0.64 | 2.95 | 2.35 | 3.55 |
| Yemen | 0.70 | 0.69 | 0.71 | 0.13 | 0.08 | 0.18 | 1.20 | 1.11 | 1.30 |
| Zambia | 0.42 | 0.41 | 0.43 | 0.53 | 0.50 | 0.57 | 4.28 | 3.78 | 4.77 |
| Zimbabwe | 0.67 | 0.66 | 0.69 | 0.40 | 0.35 | 0.45 | 1.89 | 1.71 | 2.06 |

Antenatal care quality defined as the proportion of women who report blood pressure monitoring and urine and blood testing at any point during the pregnancy among those who had at least one visit with a skilled provider.

## Appendix 5. Educational-related inequalities in antenatal care quality in 91 low and middle-income countries

| Country | Slope index of inequality in antenatal care quality | 95% CI | | Relative index of inequality in antenatal care quality | 95% CI | |
|---|---|---|---|---|---|---|
| Afghanistan | 0.14 | 0.07 | 0.20 | 1.87 | 1.31 | 2.43 |
| Albania | 0.34 | 0.24 | 0.45 | 1.57 | 1.32 | 1.82 |
| Algeria | 0.14 | 0.09 | 0.20 | 1.22 | 1.12 | 1.31 |
| Argentina | 0.03 | 0.00 | 0.05 | 1.03 | 1.00 | 1.06 |
| Armenia | 0.00 | 0.00 | 0.00 | 1.00 | 1.00 | 1.00 |
| Bangladesh | 0.39 | 0.31 | 0.47 | 2.19 | 1.75 | 2.63 |
| Belarus | 0.00 | 0.00 | 0.00 | 1.00 | 1.00 | 1.00 |
| Belize | 0.00 | -0.04 | 0.04 | 1.00 | 0.96 | 1.04 |
| Benin | 0.26 | 0.22 | 0.31 | 1.41 | 1.32 | 1.50 |
| Bhutan | 0.20 | 0.13 | 0.27 | 1.26 | 1.15 | 1.37 |
| Bolivia | 0.56 | 0.51 | 0.60 | 2.64 | 2.36 | 2.92 |
| Bosnia Herzegovina | 0.05 | -0.06 | 0.17 | 1.06 | 0.93 | 1.18 |
| Burkina Faso | 0.42 | 0.37 | 0.47 | 2.12 | 1.90 | 2.34 |
| Burundi | 0.15 | 0.12 | 0.18 | 11.41 | 5.54 | 17.29 |
| Cambodia | 0.18 | 0.12 | 0.24 | 1.51 | 1.30 | 1.72 |
| Cameroon | 0.51 | 0.46 | 0.55 | 2.07 | 1.88 | 2.25 |
| Central African Rep | 0.11 | 0.03 | 0.19 | 1.17 | 1.03 | 1.30 |
| Chad | 0.14 | 0.09 | 0.20 | 1.49 | 1.26 | 1.72 |
| Colombia | 0.02 | 0.00 | 0.04 | 1.02 | 1.00 | 1.04 |
| Comoros | 0.27 | 0.18 | 0.36 | 1.42 | 1.24 | 1.61 |
| Congo | 0.16 | 0.13 | 0.19 | 1.20 | 1.15 | 1.24 |
| Costa Rica | -0.01 | -0.14 | 0.12 | 0.99 | 0.85 | 1.12 |
| Cote d'Ivoire | 0.22 | 0.16 | 0.29 | 1.40 | 1.25 | 1.55 |
| Dem. Republic of the Congo | 0.42 | 0.38 | 0.46 | 2.71 | 2.39 | 3.04 |
| Dominican Republic | 0.04 | 0.01 | 0.06 | 1.04 | 1.01 | 1.07 |
| Egypt | 0.22 | 0.19 | 0.26 | 1.36 | 1.29 | 1.43 |
| El Salvador | 0.03 | -0.01 | 0.07 | 1.03 | 0.99 | 1.07 |
| Ethiopia | 0.44 | 0.37 | 0.50 | 2.39 | 1.99 | 2.79 |
| Gabon | 0.05 | 0.01 | 0.09 | 1.06 | 1.01 | 1.10 |
| Ghana | 0.09 | 0.06 | 0.12 | 1.10 | 1.07 | 1.14 |
| Guatemala | 0.53 | 0.50 | 0.56 | 2.51 | 2.32 | 2.70 |
| Guinea | 0.48 | 0.42 | 0.54 | 2.78 | 2.32 | 3.24 |
| Guinea Bissau | 0.24 | 0.19 | 0.29 | 1.35 | 1.25 | 1.45 |
| Guyana | 0.12 | 0.05 | 0.20 | 1.15 | 1.04 | 1.25 |
| Haiti | 0.30 | 0.25 | 0.35 | 1.48 | 1.38 | 1.59 |
| Honduras | 0.27 | 0.24 | 0.30 | 1.38 | 1.32 | 1.44 |
| India | 0.36 | 0.35 | 0.37 | 1.62 | 1.59 | 1.64 |
| Indonesia | 0.11 | 0.07 | 0.15 | 1.51 | 1.29 | 1.73 |
| Iraq | 0.09 | 0.05 | 0.14 | 1.12 | 1.07 | 1.18 |
| Jamaica | 0.04 | 0.00 | 0.09 | 1.05 | 1.00 | 1.09 |
| Jordan | 0.09 | 0.05 | 0.12 | 1.10 | 1.05 | 1.14 |
| Kazakhstan | 0.00 | 0.00 | 0.01 | 1.00 | 1.00 | 1.01 |
| Kenya | 0.28 | 0.25 | 0.32 | 1.42 | 1.35 | 1.49 |
| Kosovo | 0.27 | 0.16 | 0.37 | 1.40 | 1.19 | 1.60 |
| Kyrgyzstan | 0.28 | 0.23 | 0.32 | 3.23 | 2.52 | 3.95 |
| Laos | 0.42 | 0.35 | 0.49 | 4.29 | 2.99 | 5.58 |
| Lesotho | 0.20 | 0.14 | 0.26 | 1.29 | 1.18 | 1.39 |

| | | | | | | |
|---|---|---|---|---|---|---|
| Liberia | 0.24 | 0.20 | 0.29 | 1.35 | 1.27 | 1.44 |
| Macedonia | 0.10 | 0.01 | 0.18 | 1.11 | 1.01 | 1.22 |
| Madagascar | 0.44 | 0.40 | 0.48 | 9.31 | 7.20 | 11.42 |
| Malawi | 0.11 | 0.07 | 0.15 | 1.44 | 1.25 | 1.63 |
| Maldives | 0.03 | 0.00 | 0.07 | 1.04 | 1.00 | 1.08 |
| Mali | 0.44 | 0.38 | 0.50 | 3.03 | 2.49 | 3.57 |
| Mauritania | 0.39 | 0.33 | 0.44 | 1.82 | 1.64 | 2.00 |
| Mexico | 0.13 | 0.07 | 0.19 | 1.15 | 1.07 | 1.24 |
| Moldova | 0.01 | -0.02 | 0.04 | 1.01 | 0.98 | 1.04 |
| Mongolia | 0.09 | 0.05 | 0.12 | 1.09 | 1.05 | 1.14 |
| Montenegro | 0.01 | -0.05 | 0.08 | 1.02 | 0.94 | 1.09 |
| Mozambique | 0.26 | 0.22 | 0.31 | 2.02 | 1.75 | 2.30 |
| Namibia | 0.06 | 0.03 | 0.10 | 1.07 | 1.03 | 1.11 |
| Nepal | 0.50 | 0.44 | 0.56 | 3.79 | 3.01 | 4.57 |
| Niger | 0.43 | 0.38 | 0.47 | 4.85 | 3.89 | 5.81 |
| Nigeria | 0.41 | 0.38 | 0.44 | 1.83 | 1.72 | 1.93 |
| Pakistan | 0.66 | 0.62 | 0.70 | 5.20 | 4.34 | 6.07 |
| Palestine | 0.02 | -0.01 | 0.04 | 1.02 | 0.99 | 1.05 |
| Panama | 0.18 | 0.10 | 0.27 | 1.23 | 1.10 | 1.35 |
| Peru | 0.32 | 0.29 | 0.36 | 1.50 | 1.43 | 1.58 |
| Philippines | 0.48 | 0.43 | 0.52 | 2.52 | 2.26 | 2.78 |
| Rwanda | 0.21 | 0.16 | 0.25 | 1.48 | 1.34 | 1.62 |
| Saint Lucia | 0.00 | 0.00 | 0.00 | 1.00 | 1.00 | 1.00 |
| Sao Tome | 0.06 | 0.00 | 0.12 | 1.07 | 1.00 | 1.14 |
| Senegal | 0.27 | 0.20 | 0.35 | 1.43 | 1.29 | 1.58 |
| Serbia | -0.02 | -0.15 | 0.10 | 0.98 | 0.84 | 1.11 |
| Sierra Leone | 0.24 | 0.19 | 0.28 | 1.40 | 1.30 | 1.50 |
| South Sudan | 0.18 | 0.09 | 0.27 | 1.94 | 1.26 | 2.61 |
| Sudan | 0.40 | 0.35 | 0.45 | 1.74 | 1.59 | 1.88 |
| Suriname | -0.05 | -0.12 | 0.02 | 0.95 | 0.88 | 1.02 |
| Swaziland | 0.20 | 0.15 | 0.26 | 1.28 | 1.18 | 1.37 |
| Tajikistan | 0.09 | 0.04 | 0.14 | 1.11 | 1.04 | 1.17 |
| Tanzania | 0.47 | 0.43 | 0.51 | 2.65 | 2.36 | 2.94 |
| Thailand | 0.01 | -0.02 | 0.03 | 1.01 | 0.98 | 1.03 |
| Timor Leste | 0.06 | 0.03 | 0.09 | 1.98 | 1.22 | 2.73 |
| Togo | 0.14 | 0.09 | 0.19 | 1.18 | 1.11 | 1.25 |
| Tunisia | 0.01 | -0.06 | 0.08 | 1.01 | 0.93 | 1.09 |
| Turkmenistan | 0.50 | -0.19 | 1.20 | 2.01 | -0.81 | 4.83 |
| Uganda | 0.28 | 0.23 | 0.33 | 5.04 | 3.59 | 6.49 |
| Ukraine | 0.01 | 0.00 | 0.03 | 1.01 | 1.00 | 1.03 |
| Vietnam | 0.42 | 0.33 | 0.51 | 2.20 | 1.74 | 2.66 |
| Yemen | 0.09 | 0.04 | 0.14 | 2.20 | 1.74 | 2.66 |
| Zambia | 0.38 | 0.34 | 0.42 | 2.66 | 2.36 | 2.96 |
| Zimbabwe | 0.35 | 0.29 | 0.40 | 1.73 | 1.56 | 1.90 |

Antenatal care quality defined as the proportion of women who report blood pressure monitoring and urine and blood testing at any point during the pregnancy among those who had at least one visit with a skilled provider.

## Appendix 6. Antenatal care quality in the richest wealth quintile of 91 low and middle-income countries

| Country | Antenatal care quality |
|---|---|
| Afghanistan | 29.5 |
| Albania | 97.6 |
| Algeria | 78.1 |
| Argentina | 99.7 |
| Armenia | 100.0 |
| Bangladesh | 77.1 |
| Belarus | 100.0 |
| Belize | 100.0 |
| Benin | 92.9 |
| Bhutan | 98.7 |
| Bolivia | 90.5 |
| Bosnia Herzegovina | 98.1 |
| Burkina Faso | 82.6 |
| Burundi | 17.6 |
| Cambodia | 52.9 |
| Cameroon | 96.2 |
| Central African Rep | 79.6 |
| Chad | 59.5 |
| Colombia | 97.1 |
| Comoros | 90.9 |
| Congo | 98.7 |
| Costa Rica | 82.4 |
| Cote d'Ivoire | 84.4 |
| Dem. Republic of the Congo | 81.8 |
| Dominican Republic | 98.5 |
| Egypt | 88.2 |
| El Salvador | 97.9 |
| Ethiopia | 80.0 |
| Gabon | 96.7 |
| Ghana | 99.4 |
| Guatemala | 92.0 |
| Guinea | 87.7 |
| Guinea Bissau | 96.5 |
| Guyana | 100.0 |
| Haiti | 94.2 |
| Honduras | 97.1 |
| India | 95.1 |
| Indonesia | 33.2 |
| Iraq | 87.4 |
| Jamaica | 100.0 |
| Jordan | 96.6 |
| Kazakhstan | 100.0 |
| Kenya | 96.9 |
| Kosovo | 92.7 |
| Kyrgyzstan | 49.1 |
| Laos | 55.6 |
| Lesotho | 88.5 |
| Liberia | 96.0 |
| Macedonia | 95.6 |
| Madagascar | 61.7 |
| Malawi | 39.4 |
| Maldives | 95.8 |
| Mali | 73.5 |

| | |
|---|---|
| Mauritania | 90.0 |
| Mexico | 95.8 |
| Moldova | 98.5 |
| Mongolia | 97.7 |
| Montenegro | 99.1 |
| Mozambique | 58.5 |
| Namibia | 96.0 |
| Nepal | 77.1 |
| Niger | 65.3 |
| Nigeria | 92.9 |
| Pakistan | 83.4 |
| Palestine | 93.5 |
| Panama | 97.2 |
| Peru | 96.6 |
| Philippines | 82.7 |
| Rwanda | 71.7 |
| Saint Lucia | 100.0 |
| Sao Tome | 100.0 |
| Senegal | 92.1 |
| Serbia | 93.7 |
| Sierra Leone | 88.2 |
| South Sudan | 40.3 |
| Sudan | 95.5 |
| Suriname | 96.3 |
| Swaziland | 93.1 |
| Tajikistan | 97.9 |
| Tanzania | 85.9 |
| Thailand | 98.8 |
| Timor Leste | 11.7 |
| Togo | 96.0 |
| Tunisia | 94.1 |
| Turkmenistan | 99.6 |
| Uganda | 35.6 |
| Ukraine | 99.1 |
| Vietnam | 82.5 |
| Yemen | 76.9 |
| Zambia | 68.9 |
| Zimbabwe | 82.7 |

Antenatal care quality defined as the proportion of women who report blood pressure monitoring and urine and blood testing at any point during the pregnancy among those who had at least one visit with a skilled provider.

**Appendix 7. Antenatal care coverage and quality (measured by three and four items) by GDP per capita in 55 low and middle-income countries**

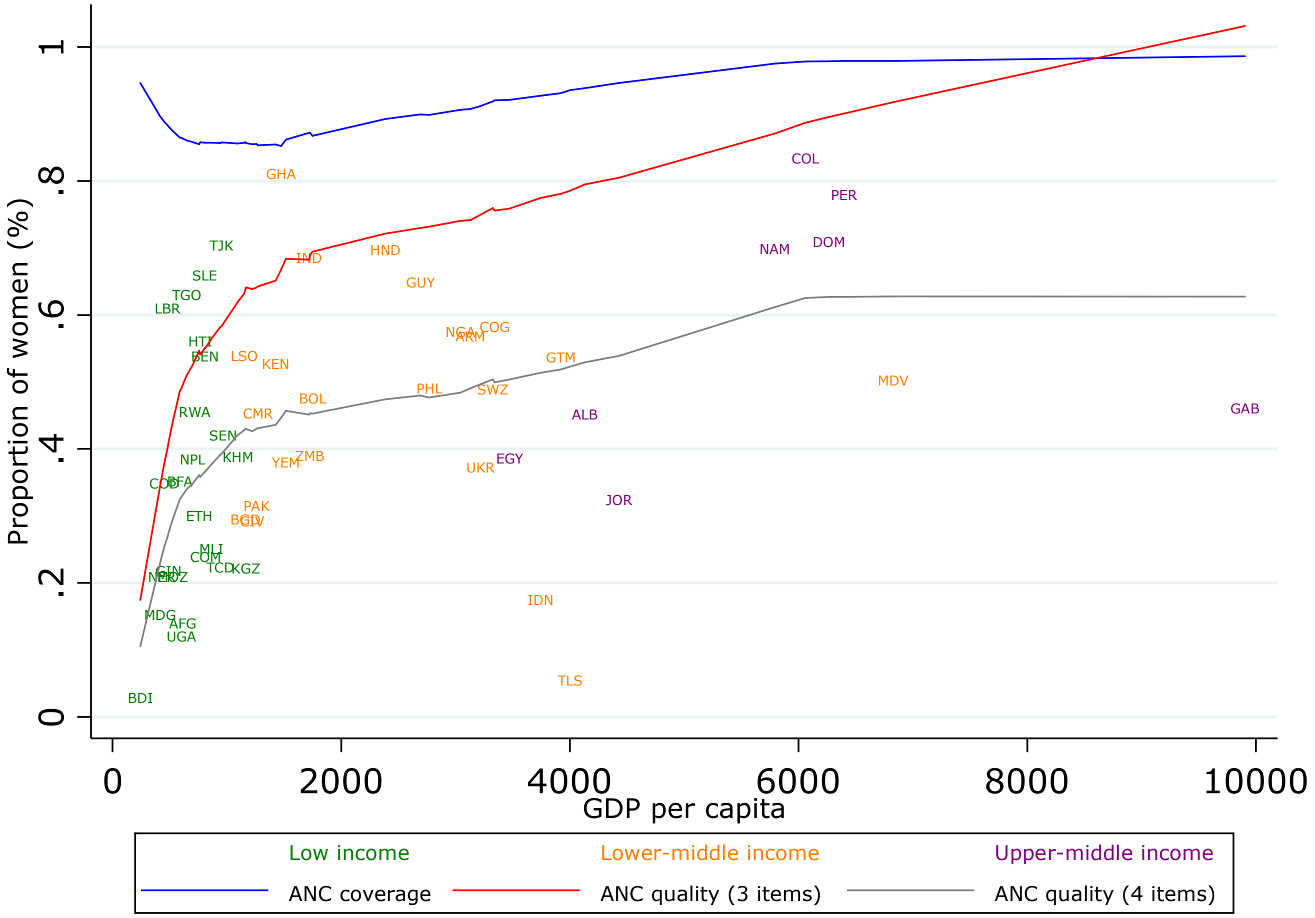

Supplement: Supplementary appendix [file mmc1.pdf]
